# Homeobox Genes and Melatonin Synthesis: Regulatory Roles of the Cone-Rod Homeobox Transcription Factor in the Rodent Pineal Gland

**DOI:** 10.1155/2014/946075

**Published:** 2014-04-30

**Authors:** Kristian Rohde, Morten Møller, Martin Fredensborg Rath

**Affiliations:** Department of Neuroscience and Pharmacology, Faculty of Health and Medical Sciences, University of Copenhagen, Rigshospitalet 6102, Blegdamsvej 9, 2100 Copenhagen, Denmark

## Abstract

Nocturnal synthesis of melatonin in the pineal gland is controlled by a circadian rhythm in arylalkylamine N-acetyltransferase (AANAT) enzyme activity. In the rodent, *Aanat* gene expression displays a marked circadian rhythm; release of norepinephrine in the gland at night causes a cAMP-based induction of *Aanat* transcription. However, additional transcriptional control mechanisms exist. Homeobox genes, which are generally known to encode transcription factors controlling developmental processes, are also expressed in the mature rodent pineal gland. Among these, the cone-rod homeobox (CRX) transcription factor is believed to control pineal-specific *Aanat* expression. Based on recent advances in our understanding of *Crx* in the rodent pineal gland, we here suggest that homeobox genes play a role in adult pineal physiology both by ensuring pineal-specific *Aanat* expression and by facilitating cAMP response element-based circadian melatonin production.

## 1. Introduction

Homeobox genes encode a large group of transcription factors involved in developmental processes throughout the animal kingdom [1]. The homeobox genes are molecularly characterized by the presence of a 180-nucleotide sequence element, the homeobox, which encodes a 60-amino acid structural motif, the homeodomain. The homeodomain recognizes specific DNA binding sites and thereby enables the homeodomain proteins to function as transcription factors [2–6]. In the pineal gland, a number of homeobox genes control developmental processes. However, certain homeobox genes predominantly exert their function in mature pinealocytes [7]. Among these, the cone-rod homeobox (*Crx*) gene seems to play an important role in transcriptional regulation of arylalkylamine N-acetyltransferase (*Aanat*) encoding the enzyme that controls the huge nocturnal peak in pineal melatonin synthesis [8].

In this paper, we review recent progress in our understanding of the biology of the CRX homeodomain transcription factor in the rodent pineal gland and propose a revised working model for the function of homeodomain transcription factors in regulation of rodent pineal* Aanat* transcription.

## 2. Homeobox Genes Regulate Pineal Gland Development

**Figure 1:**
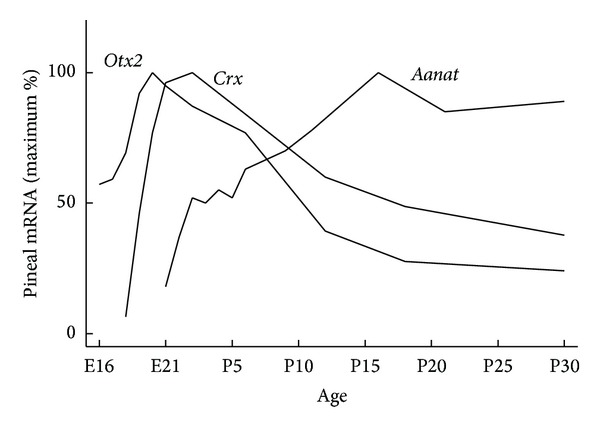
Appearance of* Otx2*,* Crx,* and* Aanat* transcripts in the developing rat pineal gland.* Otx2* and* Crx* expression peak around birth [14], at which time* Aanat* expression is initiated [19]. The spatial and temporal correlation between peaks in expression of* Otx2* and* Crx* and the start of* Aanat* expression supports that the OTX2 and CRX homeodomain transcription factors induce* Aanat* transcription [7]. E, embryonic day; P, postnatal day.

The mammalian pineal gland develops as a dorsal evagination from the most caudal part of the diencephalic roof [9]. As in other parts of the brain, timely and spatially controlled expression of a specific set of homeobox genes is essential for development of the pineal gland [7]. Gene knockout studies have identified a limited number of homeobox genes that are required for normal development of the rodent pineal gland; these include orthodenticle homeobox 2 (*Otx2*) [10], paired box 6 (*Pax6*) [11], brain specific homeobox (*Bsx*) [12], and LIM homeobox 9 (*Lhx9*) [13]. In line with a role of this set of homeobox genes in the immature developing pinealocyte, that is, the principal melatonin-producing cell type of the pineal gland,* in situ* hybridization analyses have revealed that these homeobox genes are highly expressed during early stages in rodent pineal gland development before the appearance of pineal melatonin synthesis [13–17]. However, pineal expression of homeobox genes is not restricted to prenatal stages (Figure 1); expression of homeobox genes involved in pineal development may persist into adulthood (e.g.,* Otx2*) [14], expression may start at perinatal stages just prior to the onset of melatonin synthesis (e.g.,* Crx* and retina and anterior neural fold homeobox (*Rax*)) [14, 18], or expression levels may start to increase even after or concomitant with the ontogenetic onset of melatonin synthesis (e.g.,* Pax4* and* Lhx4*) [13, 15]. Thus, another set of homeobox genes, including* Crx*, predominantly exert their function in the mature melatonin-producing pinealocyte.

## 3. Circadian Regulation of Melatonin Synthesis in the Mammalian Pineal Gland

The mammalian melatonin rhythm generating system transforms the ambient lighting conditions into the internal hormonal message of melatonin, which is restricted to night time. The system is comprised of three parts: the suprachiasmatic nucleus (SCN) of the hypothalamus, the retina, and the pineal gland [20] as opposed to the melatonin rhythm generating system in nonmammalian vertebrate species, in which the three elements are integrated into one cell [21] (see below). The SCN of mammals generates a circadian rhythm. This rhythm is established by a cellular clock mechanism that comprises interacting transcriptional feedback loops [22]. The clock mechanism of the SCN is then synchronized with the successions of day and night via specific retinal photoreceptors projecting light information to the SCN via the retinohypothalamic tract [23]. Interestingly, a subset of retinal ganglion cells contains the photopigment melanopsin, which equips the cells with light-sensing properties. These irradiance detectors integrate their information on the surrounding lighting condition with signals from rod and cone photoreceptors to photoentrain the endogenous rhythm of the SCN [24]. The circadian clock of the SCN controls the pineal gland through a multisynaptic pathway [25]. The last neuron in the pathway, which has its soma situated in the superior cervical ganglion of the sympathetic nervous system, releases norepinephrine (NE) in the perivascular spaces of the pineal gland at night. NE binds to adrenergic receptors on the membrane of the pinealocyte and activates intracellular signaling pathways; this results in nocturnal melatonin synthesis [20, 26].

The enzymes AANAT and acetylserotonin O-methyltransferase (ASMT) catalyze the conversion of serotonin to melatonin. Nocturnal increase in AANAT enzymatic activity, as a result of NE release, determines the circadian melatonin production of the pineal gland. In the rodent, a marked nightly increase of* Aanat* transcription and posttranslational modifications of the AANAT protein account for the increase in AANAT enzymatic activity [8, 27, 28]. In other mammalian species, the dynamics of the AANAT enzymatic activity is effectuated through mechanisms other than transcriptional regulation of the* Aanat* gene. For instance, in sheep, monkey, and human, pineal* Aanat* transcript levels do not oscillate on a circadian basis. However, in all mammalian species, the rhythm of pineal gland melatonin production is reliant on the nocturnal release of NE in the gland [29–31]. In the rodent, the release of NE from the sympathetic nerve endings binds and activates adrenergic receptors on the pinealocyte, which results in an increased intracellular cAMP level. cAMP-activated protein kinase A phosphorylates the cAMP response element binding protein (CREB), which binds cAMP responsive element (CRE)* cis*-regulatory elements in the* Aanat* promoter and induces transcription [32, 33]. Thus, in the rodent, transcriptional regulation of* Aanat* is of special importance for melatonin to function as the hormonal messenger of darkness.

Because cAMP does not elevate the level of* Aanat* mRNA in cells other than the pinealocytes and to some extent retinal photoreceptors [34] (see below), additional regulatory mechanisms of* Aanat* expression must exist. These are thought to play a permissive or regulatory role in ensuring tissue-specific* Aanat* expression in addition to the cAMP/CRE-based transcriptional control [7, 35]. As mentioned above, several homeobox genes have been shown to be expressed in the developing and adult pineal gland of the rodent and some have also been shown to bind photoreceptor conserved elements (PCEs) that are present in the* Aanat* promoter region and to influence* Aanat* transcription.

## 4. Local Control of* Aanat* Rhythms in Photoreceptor Cells

Melatonin is also synthesized in the mammalian retina [36, 37]. Whereas circadian biology of the mammalian pineal gland is controlled by the circadian clock of the SCN, the existence of an endogenous circadian clock within the mammalian retina is evident from the persistence of the retinal melatonin rhythm in cultured retinae [38] and SCN-lesioned rats [39]. The endogenous retinal pacemaker appears to be located in photoreceptor cells [40].

Retinal* Aanat* expression is also present in photoreceptors [41, 42] and exhibits a day-night rhythm [43]. Contrary to the regulation of* Aanat* in the pineal gland, retinal* Aanat* transcription is driven directly by clock gene products; that is, the dimer consisting of circadian locomotor output cycles kaput (CLOCK) and aryl hydrocarbon receptor nuclear translocator-like (ARNTL), which are encoded by core clock genes, binds directly to* cis*-regulatory elements, so-called E-boxes, in the* Aanat* promoter to drive circadian gene expression [44, 45]. As reviewed below, a set of homeobox genes predominantly expressed in the pinealocyte and the retinal photoreceptor seems to control tissue-specific* Aanat* expression; however, certain E-box sequences have also been shown to confer tissue specificity presumably by silencing ectopic* Aanat* expression [46]. Retinal melatonin appears to play a paracrine role in dark-adaptation processes [34] and retinal AANAT further acts to counteract light-induced retinal degeneration [47].

A close relationship between photoreception and melatonin synthesis is also seen in pineal organs of nonmammalian vertebrates, which contain cells endowed with light-sensing properties [48, 49], an endogenous circadian clock [50–52], and nocturnal melatonin production guided by daily oscillations in AANAT activity [53, 54]. From an evolutionary point of view, a common ancestral photoreceptor cell appears to have evolved into the retinal photoreceptor of the lateral eyes and the pinealocyte of the mammalian pineal gland [55, 56] which presently share both nocturnal melatonin production and the molecular characteristic of expression of a common set of homeobox genes, including* Crx*.

## 5. *Crx* Expression in the Rodent Pineal Gland

The* Crx* gene is expressed specifically in the photoreceptors of the retina and the pinealocytes of the pineal gland in the adult rodent [7, 14, 57–59]. During development,* Crx* is expressed in the rat pineal gland from embryonic day (E) 18 onwards with a peak around birth (Figure 1), suggesting that the role of* Crx* is not at the earliest of pineal gland developmental stages, but rather later on when the pinealocytes become differentiated [14]. Around the same embryonic stage,* Crx* expression has been reported in rat retinal photoreceptor cells [57]. Therefore,* Crx* seems to be important when differentiation processes occur in both photoreceptors and pinealocytes.* Otx2* is expressed in the fore- and midbrain regions in the developing central nervous system. The total* Otx2*-knockout mouse lacks the forebrain and midbrain regions at embryonic developmental stages. However, in a mouse with conditional* Otx2* gene knockout specifically in the developing photoreceptors and pinealocytes, it has been shown that* Otx2* is essential for development of retinal photoreceptors and pinealocytes [10, 60, 61]. Further, OTX2 transactivates* Crx* expression [10] and this is in accord with the timing of the developmental peak of* Otx2* in both photoreceptors and pinealocytes, which precedes that of* Crx* (Figure 1) [14, 57].

**Figure 2:**
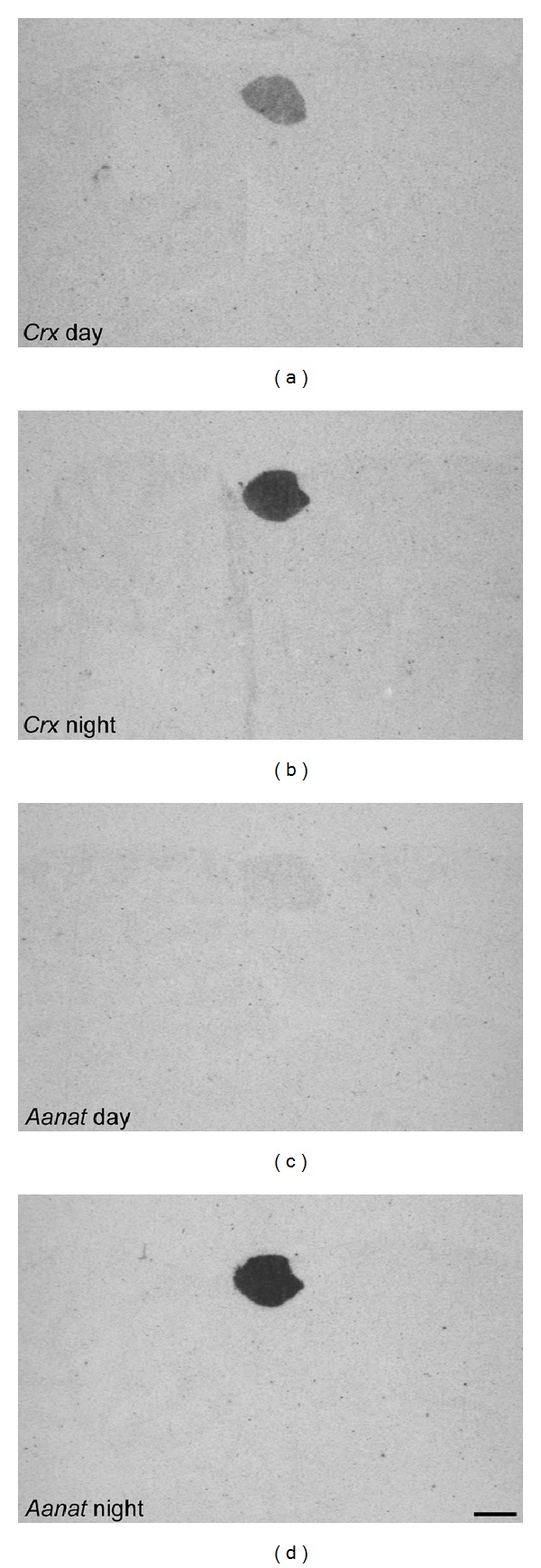
*Crx* and* Aanat* day/night expression in the adult rat pineal gland. Radiochemical* in situ *hybridization autoradiographs of midsagittal brain sections through the pineal gland of rats killed at daytime (Zeitgeber time 6) and nighttime (Zeitgeber time 18). Images are orientated with caudal to the left.* Crx* displays a specific expression in the pineal gland. Studies have shown a diurnal rhythm of* Crx* expression in the rat pineal gland with a night time peak [7, 35, 71].* Aanat* also exhibits a pineal-specific expression; however, daytime transcript levels are very low. Scale bar, 1 mm. Methodological details have been previously published [14, 71].

Expression of* Aanat* and* Asmt* in the developing rat pineal gland starts around birth [19, 62]. Interestingly, several homeobox genes display a pineal expression peak around the appearance of* Aanat* and* Asmt *and expression persists into adulthood, suggesting that these homeodomain transcription factors are important for inducing expression of the enzymes required for melatonin synthesis and thereby ensuring a pineal-specific circadian melatonin output (Figure 1). Persistent ontogenetic expression of pineal homeobox genes is seen in the case of* Otx2 *and* Crx *(Figure 2) [14], as well as* Rax* [18, 63]. The first* Rax* gene expression is confined to the forebrain and midbrain region of the developing central nervous system, but later becomes progressively restricted to specific brain areas, including the pineal gland and neural retina [64, 65]. Moreover, RAX protein also plays a role in the transcriptional regulation of the* Otx2* gene through a newly discovered* cis*-regulatory enhancer region in the* Otx2* gene that RAX is capable of transactivating in photoreceptor precursors [66]. Notably, this enhancer sequence is also active in the rodent pineal gland at postnatal stages [67]. On the contrary, a classical homeobox gene such as* Pax6* that is widely expressed in the developing central nervous system and is required for a normal development of the pineal gland [11, 68] is nearly undetectable in the pineal gland of the adult rodent [15]. In the mouse retina, PAX6 is a suppressor of* Crx* expression during development, thereby preventing an onset of premature photoreceptor differentiation [69]. The concurrent decline of* Pax6* expression and increase of* Crx* expression in developing rat pinealocytes may reflect a similar functional relationship between these two transcription factors, permitting CRX to exert its control over transcription of the melatonin synthesizing enzymes and pinealocyte differentiation [7]. Complete maturation of pineal physiology with circadian melatonin synthesis occurs in the second postnatal week, at the time when sympathetic innervation of the gland is established and the SCN is capable of exerting its circadian control of the pineal endocrine output [7, 70].

## 6. Roles of CRX in the Rodent Pineal Gland

Investigations in a* Crx*-knockout mouse have demonstrated a central role of* Crx* in the developing rodent visual system. Elimination of* Crx* causes a lack of photoreceptor outer segments, a reduced expression of several photoreceptor-specific genes accompanied by a disturbed development, and phenotype of different neuronal populations in the primary visual cortex. Further, circadian entrainment is attenuated [73, 74]; temperature and running diurnal rhythms display decreased robustness [75]. The morphology of the pineal gland appears normal [76]; however, the expression of pineal* Aanat* is reduced [73, 75, 76].

The observed influence of* Crx* on expression of several photoreceptor- and pineal-specific genes is in accord with the presence of* cis*-regulatory PCEs in the promoter region of these genes, including the* Aanat* promoter [35].* In vitro* studies have shown that the CRX protein binds PCEs and causes transactivation of reporter constructs [35, 58, 59, 77, 78]. Notably, the promoter of the neural retina leucine zipper (*Nrl*) gene, which is a basic-leucine zipper (bZIP) transcription factor [79], drives tissue-specific expression in rod photoreceptors and the pineal gland [80]. NRL and CRX have been shown to transactivate the rhodopsin promoter in a synergistic manner, and the rhodopsin gene is also expressed in the mammalian pineal gland [58, 81]. Thus, a similar cooperation between NRL and CRX is possible in the context of transcriptional regulation of* Aanat*, since both the* Aanat* and rhodopsin gene contain the PCE regulatory element in their promoter region [77]. In the adult rat pineal gland, many of the homeobox genes, which show a developmental expression peak around the time of the appearance of* Aanat* and* Asmt* expression, also display a diurnal rhythm of expression, that is,* Rax*,* Otx2,* and* Crx* [7, 18]. During the 24 h period, expression levels of these homeobox genes peak in a sequential manner before the late night expression peak of* Aanat*. That is,* Rax* peaks in the transition from day to night,* Otx2* early in the night, and* Crx* in the middle of the night [7]. The daily expression profiles, existing data on binding of RAX, OTX2, and CRX homeodomain proteins to PCEs and the transactivating property of RAX on* Otx2* transcription and of OTX2 on* Crx* transcription [10, 35, 58, 59, 66, 77, 78, 82], suggest that homeodomain proteins play a role in the expression of genes like* Aanat* that otherwise show a CRE-based circadian rhythm [32]. Thus, the occurrence of the* Crx* expression peaks a few hours before the peak of* Aanat* expression additionally supports the concept that homeodomain proteins act as regulatory factors of the daily expression profile of* Aanat* in the rodent pineal gland with CRX in a central position.

## 7. *Crx* in the Retinal Photoreceptor and Nonmammalian Pinealocyte

Photoreceptors are present in the retina of all vertebrates and in the pineal gland of most nonmammalian vertebrates [83]. In the retinal photoreceptor,* Crx* is thought to be involved in the process of terminal differentiation of photoreceptors and is essential for formation of photoreceptor outer segments, as evidenced by gene knockout studies [73, 84]. At the molecular level, CRX is centrally placed in a network of transcription factors that regulate photoreceptor gene expression and thereby terminal differentiation of the various photoreceptor subtypes [85, 86]. As in the rodent pineal gland, expression of* Crx* persists in the mature retina [14, 57–59], where CRX seems to positively control expression of a number of genes involved in phototransduction [58, 59, 87, 88]. Many of these genes are also detectable in the mammalian pineal gland, reflecting the common phylogenetic relationship between the retinal photoreceptor and the pinealocyte.

In contrast to the mammalian pineal gland, the zebrafish pineal organ comprises cells that are capable of photodetection, which enable entrainment of the endogenously generated rhythm that controls circadian expression of genes in pinealocytes and hence the nocturnal synthesis of melatonin [89]. In zebrafish, the* Otx5* gene, which is orthologous to the mammalian* Crx* gene, regulates genes that exhibit a circadian pattern of expression in the pineal complex. A lack of OTX5 protein attenuates the circadian pineal expression of* Aanat2*, which is the homolog of the mammalian* Aanat* gene and thus encodes the rate limiting enzyme in the zebrafish pineal production of melatonin [90]. Similar to the role of CRX in mammals, the action of OTX5 on* Aanat2* in the zebrafish pineal organ is mediated through PCEs [77, 91, 92]. Interestingly, in the zebrafish pineal gland, OTX5, in addition to ensuring pineal-specific expression of* Aanat2*, is also capable of facilitating the circadian expression of* Aanat2*, which, in accordance with the situation in the mammalian retina, is otherwise controlled by the circadian CLOCK/ARNTL-dimer [91].

Like the pinealocyte of the zebrafish, the chicken pinealocytes possess the capability of light perception [48]. It has been shown in chicken that CRX activates transcription of* Asmt* through the interaction with PCEs in the* Asmt* promoter region [93]. A similar relationship has been shown between chicken* Asmt* and OTX2 [94]. Thus, CRX and the related OTX2 seem to exert a highly conserved regulatory role in transcriptional regulation of the enzymes involved in melatonin synthesis between vertebrate species.

## 8. A Proposed Model for Shaping the Daily* Aanat* Expression Profile

**Figure 3:**
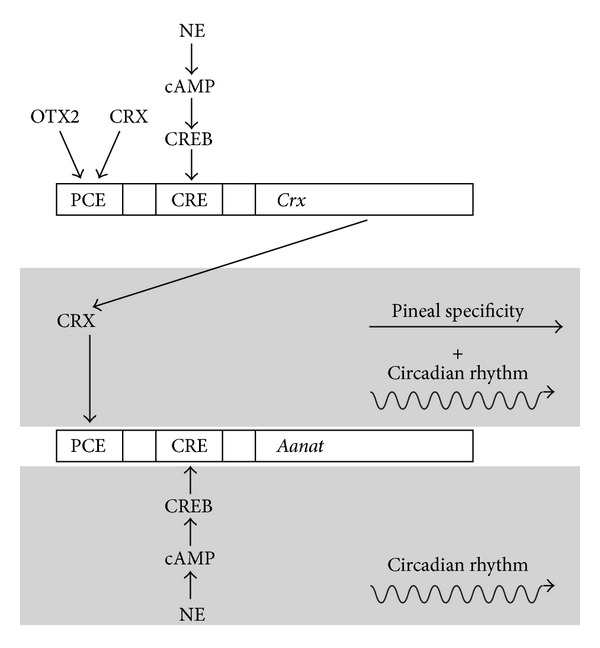
Working model of the role of homeobox genes in generation of pineal specificity and circadian output. CRX and OTX2 homeodomain transcription factors and NE/cAMP/CREB signaling, initiated by NE released from the sympathetic nerve endings at night, act on PCE and CRE* cis*-regulatory elements in the pineal* Crx* promoter region, respectively, to generate tissue-specific circadian expression of* Crx*. CRX protein acts on PCEs in the* Aanat* promoter region and confers a pineal-specific and circadian expression of* Aanat*. The homeodomain transcription factor generated rhythmicity of* Aanat* transcription supports the classical NE/cAMP/CREB-driven circadian rhythm of* Aanat* expression. The* Crx* promoter contains at least one PCE and several CRE* cis*-regulatory elements. In the* Aanat* promoter, several CREs and at least two PCEs exist. Promoter analysis was performed using Genomatix MatInspector software [72]. CRE, cAMP response element; CREB, cAMP response element binding protein; PCE, photoreceptor conserved element.

By the use of adenovirus-mediated shRNA knockdown and overexpression of* Crx* mRNA in cultured rat pinealocytes and investigations performed in the* Crx*-knockout mouse, it has been shown that CRX induces* Aanat* transcription in the rodent. At the same time, CRX exhibits a circadian rhythm in the pineal gland driven by the nocturnal release of NE from sympathetic nerve endings in the gland (Rohde K, Rovsing L, Ho AK, Møller M, and Rath MF, in preparation). Based on these findings and existing data on* Aanat* transcription, we here propose an extended working model for the role of homeodomain transcription factors in generation of pineal specificity and circadian output (Figure 3). Thus, in addition to ensuring pineal specificity of* Aanat* expression, mammalian CRX may also facilitate daily changes in* Aanat* expression and therefore act to shape the daily profile of melatonin synthesis.

## Abbreviations

*Aa**na**t*: Arylalkylamine N-acetyltransferase
*Ar**nt**l*: Aryl hydrocarbon receptor nuclear translocator-like
*As**mt*: Acetylserotonin O-methyltransferase
*Bs**x*: Brain specific homeobox
bZIP: Basic-leucine zipper
*Cl**oc**k*: Circadian locomotor output cycles kaput
CRE: cAMP response element
CREB: cAMP response element binding protein
*Cr**x*: Cone-rod homeobox
E: Embryonic day
*Lh**x*: LIM homeobox
NE: Norepinephrine
*Nr**l*: Neural retina leucine zipper
*Ot**x*: Orthodenticle homeobox
P: Postnatal day
*Pa**x*: Paired box
PCE: Photoreceptor conserved element
*Ra**x*: Retina and anterior neural fold homeobox
SCN: Suprachiasmatic nucleus.
